# Enteric Delayed-Release Granules Loading Dendrobine Ameliorates Hyperlipidemia in Mice by Regulating Intestinal Flora Composition

**DOI:** 10.3390/pharmaceutics16111483

**Published:** 2024-11-20

**Authors:** Shunqiang Song, Liangyu Yang, Tingting Chen, Yongai Xiong

**Affiliations:** 1 Key Laboratory of Basic Pharmacology of Guizhou Province and School of Pharmacy, Zunyi Medical University, Zunyi 563000, China; 2 Key Laboratory of Basic Pharmacology of Ministry of Education and Joint International Research Laboratory of Ethnomedicine of Ministry of Education, Zunyi Medical University, Zunyi 563000, China

**Keywords:** hyperlipidemia, intestinal flora, Dendrobine, enteric-coated delayed-release granules

## Abstract

**Background/Objectives**: In this paper, we created enteric delayed-release granules that load Dendrobine (DNL) directly into the intestinal flora of hyperlipidemic mice, based on the relationship between intestinal flora and hyperlipidemia. **Methods**: We then used pharmacodynamics and 16 Sr RNA high-throughput sequencing to examine the hypolipidemic effects and mechanism of these granules. Solvent evaporation was used to create the DNL, which was then characterized using FT–IR, XRD, SEM, and DSC. A high-fat diet was used to create the mouse model of hyperlipidemia in C57BL/6J mice. Dendrobine, various dosages of DNL, TMAO, and the combination of TMAO and DNL were subsequently gavaged on the mice. The makeup of the intestinal flora in the mouse colon was analyzed using 16S rRNA sequencing, and the effectiveness and mechanism of DNL in controlling the intestinal flora for the treatment of hyperlipidemia in mice were investigated. **Results/Conclusions**: The findings showed that DNL could effectively improve the dysbiosis brought on by hyperlipidemia by significantly lowering the mice’s body weight and blood lipid level (*p* < 0.05), while also regulating the function of their intestinal flora, increasing the abundance of Actinobacteria (*p* < 0.05) and Thick-walled bacterium (*p* < 0.05), and decreasing the abundance of Desulfovibrio (*p* < 0.05) and Mycobacterium anisopliae (*p* < 0.05) in the intestinal flora of mice, inhibiting the growth of intestinal harmful microorganisms, providing space for the reproduction of beneficial bacteria, and thus maintaining the stability of the intestinal flora’s structure.

## 1. Introduction

Hyperlipidemia, a disorder of the body’s lipid metabolism, causes blood lipid levels to rise. This is one of the major causes of atherosclerosis, which has been dubbed the “silent killer” and is an independent risk factor for a number of cardiovascular and cerebrovascular diseases, including ischemic stroke and coronary atherosclerotic heart disease [1]. According to recent research, 40% of patients still have residual cardiovascular risk even after statin treatment, and even if low-density lipoprotein cholesterol (LDL-C) drops to levels advised by guidelines. As a result, clinical patients urgently need safer and more effective lipid-lowering medications.

Recent studies have found a strong association between gut flora, high-fat diet and hyperlipidemia [2]. Intestinal flora may play a role in lipid regulation by producing cholesterol oxidase, inhibiting hepatic lipo-synthesis enzyme activity, regulating the redistribution of cholesterol in the blood and liver, and influencing the hepatic and intestinal circulation of bile salts [3]. The molecular mechanisms by which intestinal flora regulate lipid metabolism include the direct regulation of host fat storage genes (inhibition of fasting-induced adipocytokine gene activity, enhancement of the expression of sterol response element-binding protein 21 and carbohydrate response element-binding protein, reduction of phosphorylated adenylyl acid-activated protein kinase activity, and modulation of lipopolysaccharide activity in order to alter the body’s chronic low-level inflammatory state and influence lipid metabolism [4,5]). Therefore, inhibiting the formation of hyperlipidemia by regulating intestinal flora has become a currently important strategy to improve hyperlipidemia.

**Figure 1:**
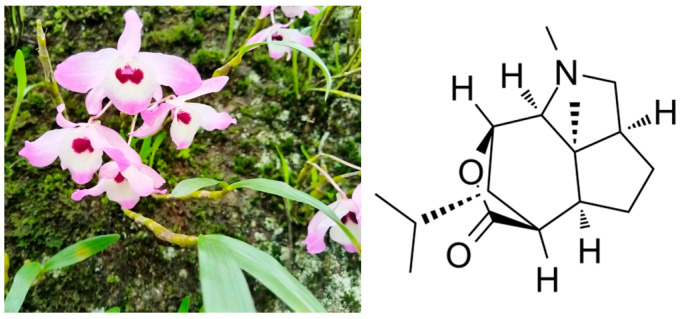
Dendrobium nobile Lindl and the chemical structure of Dendrobine.

Dendrobium nobile Lindl. is the dried stem of Dendrobium nobile, a perennial herb in the genus Dendrobium, family Orchidaceae, and is a traditional and valuable Chinese medicine [6]. Several studies have shown that extract of Dendrobium officinale has obvious hypolipidemic effects, and its main active ingredient is Dendrobine (its structure is shown in Figure 1), but its hypolipidemic mechanism of action has not yet been revealed [7,8,9]. We speculate that the hypolipidemic effect of Dendrobium officinale may be related to the regulation of intestinal flora.

In this study, based on the interaction between intestinal flora and hyperlipidemia, Dendrobine was made into enteric-coated delayed-release granules to directly target intestinal flora, and 16 SrRNA high-throughput sequencing technology was applied to study its therapeutic effect and mechanism on hyperlipidemic mice.

## 2. Materials and Methods

### 2.1. Materials

Dendrobine was purchased from MUST Biotech Co., Ltd. (Chengdu, China). Trimetlylamine oxide (TMAO), Simvastatin, and Polyacrylic acid resin II and III were purchased from Roen Biotechnology Co., Ltd. (Guangzhou, China). Total cholesterol (TC), triglyceride (TG), Low-Density Lipoprotein Cholesterol (LDL-C) and High density lipoprotein cholesterol (HDL-C) ELISA kits were purchased from Beyotime Biotechnology Co., Ltd. (Shanghai, China).

### 2.2. Animals

Tianqing Biotechnology Co., Ltd. of Changsha (Changsha, China) provided SPF grade KM mice (weighing 20 ± 2 g). During the experiment, the animals were housed individually in SPF facilities. The Ethics Committee of Zunyi Medical University approved all of the animal experimental protocols (Appl. No. ZMU21-2306-046). The Laboratory Animal Welfare and Ethics Committee of China oversaw all animal care and experimental procedures.

## 3. Methods

### 3.1. Preparation of Dendrobine-Solid Dispersions

The solid dispersion of Dendrobine (DNL-SD) was prepared by solvent method: Dendrobine and polyacrylic acid resin II/polyacrylic acid resin III were separately and precisely weighed according to the ratios 1:3, 1:5, and 1:7 (*w*/*w*) in a beaker, and 500 mL of anhydrous ethanol was added to dissolve them; they were then transferred to a round-bottomed flask after 6 h of stirring, and rotary evaporation was performed to remove solvents at a temperature of 50 °C, and the precipitates stored in the vacuum drying oven for 48 h. The two prepared DNL-SDs in parallel were then stored in a vacuum drying oven for 48 h and then removed. After 48 h of storage, the precipitate was removed, put through a 100-mesh sieve, and stored in a desiccator for further use.

### 3.2. Drug Release Test

A drug dissolution meter (RC1209, Tianda Tianfa Technology Co., Tianjin, China) was used to study the release of two DNL-SDs prepared in a 2.1 proportion. Briefly, 750 mL of 0.1 mol·L^−1^ hydrochloric acid buffer and pH 6.8 phosphate buffer were used as dissolution media, the rotational speed was 50 r·min^−1^, the temperature was 37 °C, and 5 mL of samples were taken at 1.0, 2.0, 8.0, 12.0, 24.0, 36.0, 48.0, and 72.0 h. The samples were made up at the same temperature and simultaneously, with the same volume of dissolution media. In these conditions, the sample was filtered through a 0.45 μm syringe filter, the filtrate was dried under vacuum, 1.0 mL of naphthalene internal standard solution and methanol solution were added, after which the mixture was filtered through a 0.45 μm syringe filter, and analyzed according to the chromatography conditions established previously. The peak area was recorded and the cumulative solubility was calculated.

(1) $$Q=\frac{CnV0+\sum \begin{array}{c} n-1\\ i=1 \end{array}CiVi}{m}\times 100\%$$

### 3.3. Characterization of DNL-SD

#### 3.3.1. Differential Scanning Calorimetry (DSC)

Appropriate amounts of Dendrobine, polyacrylic acid resin II, polyacrylic acid resin III, Dendrobine–polyacrylic acid resin III as a physical mixture, and DNL-SD(II) and DNL-SD(III) were weighed in an aluminum crucible, and the DSC curves were tested under the conditions of 0–350 °C and a temperature increase rate of 10 °C·min^−1^.

#### 3.3.2. X-Ray Powder Diffraction (XRD)

Appropriate amount of Dendrobine, Polyacrylate Resin II, Polyacrylate Resin III, Dendrobine-Polyacrylate Resin III Physical Mixture and DNL-SD (II), DNL-SD (III) were weighed respectively at the sample stage, then spread out and placed on the XRD instrument for testing.

#### 3.3.3. Fourier Transform Infrared Spectroscopy (FT–IR)

130 mg of potassium bromide and 1.3 mg of Dendrobine, polyacrylic acid resin II, polyacrylic acid resin III, Dendrobine-polyacrylic acid resin III physical mixture and DNL-SD (II), DNL-SD (III) were placed into an agate grinding bowl and ground to less than 2 μm, grinding in one direction and then pouring the ground samples into molds and pressing the powders into transparent flakes with a tableting machine. The powders were put into the FTIR instrument for testing.

#### 3.3.4. Microscopic Scanning Electron Microscope Observation (SEM)

VA vacuum spray plating method was used: appropriate amounts of Dendrobine, polyacrylic acid resin II, polyacrylic acid resin III, Dendrobine–polyacrylic acid resin III as a physical mixture, and DNL-SD (II), DNL-SD (III) were weighed at the sample stage, rotary activity was carried out, the gold was sprayed (10 KV for 60 s), and the uniformity of spraying and plating was observed under the microscope.

### 3.4. Preparation of Enteric Delayed-Release Granules

The enteric delayed-release granules were prepared by wet granulation method. A mixture of soluble starch and microcrystalline cellulose in a ratio of 3:2 (g/g) was used as a filler for the granules, and an ethanol solution with concentration of 50.5% was the wetting agent. The ratio of DNL-SD and filler was 1:3 (g/g), and the amount of wetting agent was 2.4%.

### 3.5. Animal Experimentation

#### 3.5.1. Mice Grouping and Administration

After 2 weeks of adaptive feeding, specific pathogen-free (SPF) KM mice were divided into three groups randomly (Normal group (NOR), Model group (MOD), Positive group (SIM), DNL low dose group (DNL-L), DNL medium dose group (DNL-M), DNL high dose group (DNL-H), Dendrobine reference group (DNL-E), trimethylamine oxide (TMAO) and TMAO + DNL)), 10 mice in each group (half male and half female). Mice in the NOR group (LFD + PBS) were fed a low-fat diet as healthy control, while mice in other groups were fed a high-fat diet for 12 weeks to construct hyperlipidemia models. Mice in the MOD group were administered PBS instead. Mice in the SIM group were treated with a daily oral gavage with 100 mg/kg of simvastatin. Mice in the DNL-L, DNL-M and DNL-H groups were treated with a daily oral gavage with 50, 100 and 150 mg/kg of enteric delayed-release granules in dissolved solution. Mice in the DNL-E group were treated with a daily oral gavage with 100 mg/kg of non-enteric Dendrobine. Mice in the TMAO group were treated with a daily oral gavage with 50 mg/kg of TMAO. Mice in the TMAO + DNL group were treated with a daily oral gavage with 50 mg/kg of TMAO and 100 mg/kg of enteric delayed-release granules in a dissolved solution. The body weight of mice in each experimental group was weighed and recorded weekly.

#### 3.5.2. Sample Collection and Processing

After 2 weeks treatment, the mice were euthanized, and blood and liver were collected and immediately stored at −80 °C. The levels of TC, TG, LDL-C, and HDL-C were detected. The liver tissues of mice were dissected, weighed, and the liver index calculated. The liver index = liver weight (mg)/body weight (g). A portion of mice liver tissues were taken and used for Hematoxylin–Eosin (HE) staining after fixation with 4% paraformaldehyde. Furthermore, the contents of the cecum of the mice were collected under anaerobic conditions, quick-frozen in liquid nitrogen, and preserved at −80 °C, the contents of the colon were subjected to 16S rRNA high-throughput sequencing performed to amplify the 6SV3-V4 amplification region, and the primer sequences used were CCTAYGGGGRBGCASCAG, GGACTACNNGGGGTATCTAAT.

Small fragment libraries were constructed according to the characteristics of the amplified regions, and these libraries were subjected to double-end sequencing based on the Illumina NovaSeq sequencing platform. After Read splicing and filtering, OTU (Operational Taxonomic Unit) clustering or ASV (Amplicon Sequence Variants) noise reduction, the validated data were subjected to species annotation and abundance analysis to reveal the species composition of the samples.

## 4. Statistical Analysis

All data were processed by SPSS 20.0 software. Data were expressed as “mean ± standard deviation”. Data results were analyzed using one-way analysis of variance (ANOVA), differences between groups were also analyzed using ANOVA, and the least significant difference method (LSD method) was used for two-by-two comparisons between groups, with *p* < 0.05 indicating that the differences were statistically significant.

## 5. Results

### 5.1. Comparison of Cumulative Drug Release Properties of Different Categories of DNL-SD

**Figure 2:**
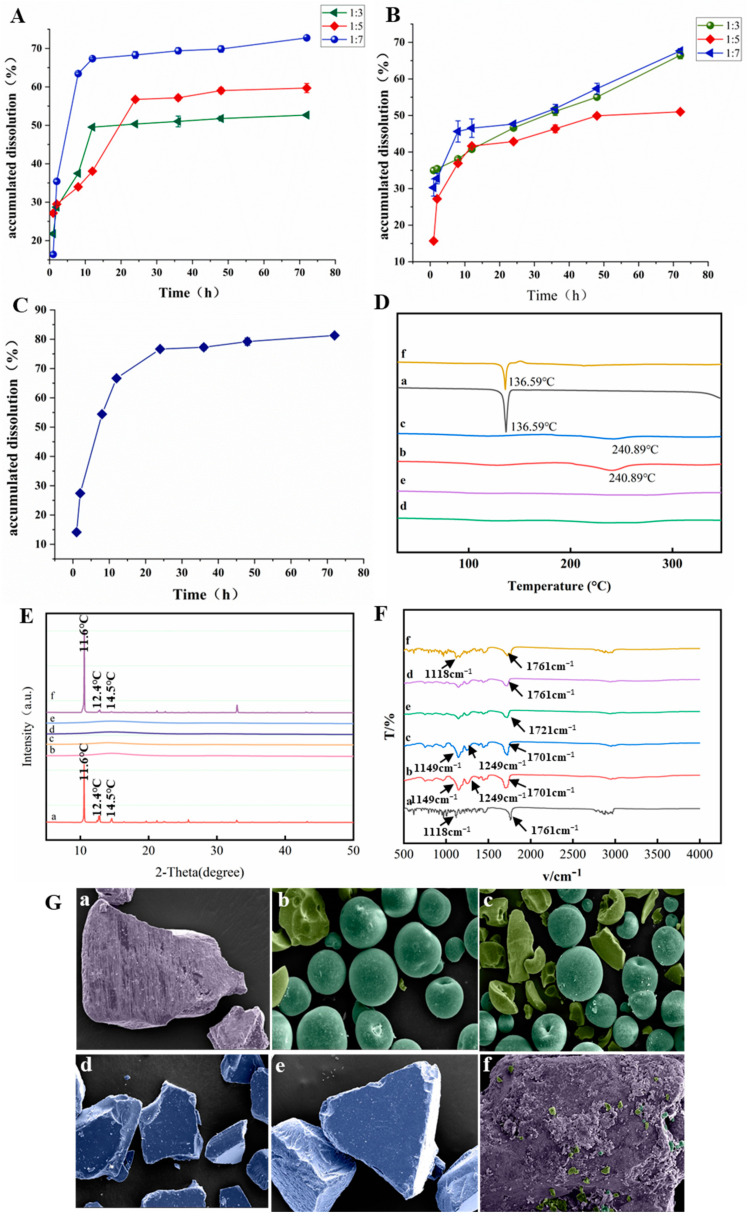
Characterization of DNL-SD. (**A**): The cumulative release of DNL-SD prepared from polyacrylic acid resin II. (**B**): The cumulative release of DNL-SD prepared from polyacrylic acid resin III. (**C**): The cumulative release of DNL-SD prepared from a mixture of excipients consisting of polyacrylic acid resin II and polyacrylic acid resin III in a 1:1 ratio (g/g) in PBS buffer. (**D**): The Differential Scanning Calorimetry thermogram. (**E**): The X-Ray Diffraction thermogram. (**F**): The Fourier Transform infrared spectroscopy spectrum. (**G**): The SEM micrographs. (**a**). Dendrobine. (**b**). Polyacrylic acid resin II. (**c**). Polyacrylic acid resin III. (**d**). DNL-SD (II). (**e**). DNL-SD (III). (**f**). Physical mixture.

We compared DNL-SDs prepared from polyacrylic acid resin II, polyacrylic acid resin III and a mixture of the two (1:1 ratio) as excipients and found that all three DNL-SDs had good acid stability, but the drug release varied greatly. The maximum cumulative release of DNL-SD made from polyacrylic acid resin II in PBS buffer for 72 h was (72.73 ± 0.13)%, as shown in Figure 2A. The maximum cumulative release of DNL-SD made from polyacrylic acid resin III in PBS buffer for 72 h was (67.65 ± 0.63)%, as shown in Figure 2B. However, the maximum cumulative release of DNL-SD prepared from a mixture of excipients consisting of polyacrylic acid resin II and polyacrylic acid resin III in a 1:1 ratio (g/g) in PBS buffer for 72 h can reach up to (81.29 ± 0.30)%, as shown in Figure 2C. The release rate profile the DNL-SD exhibits is compatible with the range of gastrointestinal times reported for mice [10]. Thus, a mixture of excipients consisting of polyacrylic acid resin II and polyacrylic acid resin III in a 1:1 ratio (g/g) was used as the optimal excipient for the preparation of the DNL-SD.

### 5.2. Dendrobine-Solid Dispersion Characterization

#### 5.2.1. Differential Scanning Calorimetry (DSC)

As shown in Figure 2D, Dendrobine has a sharp characteristic peak of heat absorption at around 169.59 °C, which shows its crystalline nature; the physical mixture of Dendrobine and poly(acrylic acid) resin III has a peak of heat absorption from at around 136 °C and, at the same time, there is a characteristic peak of heat absorption from poly(acrylic acid) resin III at around 212.62 °C, which shows that the physical mixture of Dendrobine and polyacrylic acid resin III did not change the crystalline form of Dendrobine, and the two were simply mixed; polyacrylic acid resin II had heat absorption peaks at 127.29 °C and 240.35 °C, and polyacrylic acid resin III had heat absorption characteristic peaks at 117.29 °C and 240.85 °C; DNL-SD(II) had heat absorption peaks at 122.68 °C and 237.69 °C, which were shifted compared to that of polyacrylic acid resin II and Dendrobine, and DNL-SD(II) was more suitable for the physical mixture of Dendrobine and polyacrylic acid resin III. DNL-SD(III) had heat-absorption peaks at 99.56 °C and 275.65 °C, which were shifted compared to the heat-absorption characteristic peaks of polyacrylic acid resin III and dendrobium base, suggesting that the dendrobium base may have interacted with polyacrylic acid resin II and polyacrylic acid resin III in DNL-SD(II) and DNL-SD(III), respectively, and was no longer a simple physical mixture.

#### 5.2.2. X-Ray Powder Diffraction (XRD)

As shown in Figure 2E, the more obvious characteristic diffraction peaks of Dendrobine existed at 11.6°, 12.4°, 14.5°, etc., whereas the carrier polyacrylic acid resin II and polyacrylic acid resin III did not exist at the above diffraction peaks, and neither did DNL-SD (II) and DNL-SD (III); moreover, the physical mixture of Dendrobine and polyacrylic acid resin III also displayed more obvious characteristic diffraction peaks at 11.6°, 12.4°, 14.5°, etc., suggesting that the two were simply mixed in SD, thus indicating that the two might exist in amorphous form in SD.

#### 5.2.3. Fourier Transform Infrared Spectroscopy (FT–IR)

As shown in Figure 2F, the absorption peaks at 1761 cm^−1^ in the infrared spectrum of Dendrobine are caused by -C=O stretching vibration, and the absorption peak at 1118 cm^−1^ is caused by C-O-C stretching vibration; the absorption peaks at 1701 cm^−1^ and 1149 cm^−1^ in the infrared spectrum of polyacrylic acid resin II are caused by C=O, C-O-C stretching vibration, and the absorption peak at 1249 cm^−1^, and the absorption peak at 1249 cm^−1^ are caused by C-N stretching vibration; in the infrared spectrum of polyacrylic acid resin III, the absorption peaks at 1701 cm^−1^ and 1149 cm^−1^ are caused by C=O and C-O-C stretching vibration, and the absorption peak at 1249 cm^−1^ is caused by C-N stretching vibration; the infrared spectrogram of the physical mixture of Dendrobine-polyacrylic acid resin III is a simple superposition of the signal peaks of Dendrobine and polyacrylic acid resin; the characteristic peaks, similar to those of Dendrobine, in the infrared spectrograms of DNL-SD(II) and DNL-SD(III) were shifted to 1720 cm^−1^ and 1721 cm^−1^, respectively, and the peaks’ intensities were significantly decreased, which is possibly due to the intervention of the polyacrylic acid resin and carbonyl The change in surrounding environment led to the change in peak position, the -C=O peak moved to the direction of low wave number and appeared at 1720 cm^−1^, 1721 cm^−1^, and the characteristic peaks, similar to those of polyacrylic acid resin, 1701 cm^−1^, 1149 cm^−1^, 1249 cm^−1^, were shifted to 1437 cm^−1^, 1243 cm^−1^, and 1145 cm^−1^.This indicated that Dendrobine was successfully dispersed in the polyacrylic acid resin, the solid dispersion system had been successfully prepared, and the spatial structure had changed, so the position of the absorption peaks had changed.

#### 5.2.4. Microscopic Scanning Electron Microscope Observation (SEM)

As shown in Figure 2G, Dendrobine has a crystalline bulk structure, while polyacrylic acid resin II and polyacrylic acid resin III contain methacrylic acid and methacrylate, which appear in spherical and bulk form, the physical mixture presents a simple mixed state of the two, and the polyacrylic acid resin attaches to the surface of Dendrobine, with micrometer-sized particles. This shows that DNL-SD has a rough surface and an agglomerated microstructure, which is in the form of amorphous lumps, indicating that the dosage changes the form of Dendrobium alkali so that they exist in a dispersed state, and that holes left by solvent evaporation can be seen in DNL-SD.

### 5.3. Animal Experimentation

#### 5.3.1. Morphological Observations

The mice in the NOR group were in good spirits, had normal feces, and glossy back fur. After feeding with high-fat chow, the remaining mice in each experimental group had rough fur, were fat, showed oily feces with a strong odor, had greasy, dirty, and arched backs, and appeared to be in poor spirits.

#### 5.3.2. Blood Lipid Level

**Figure 3:**
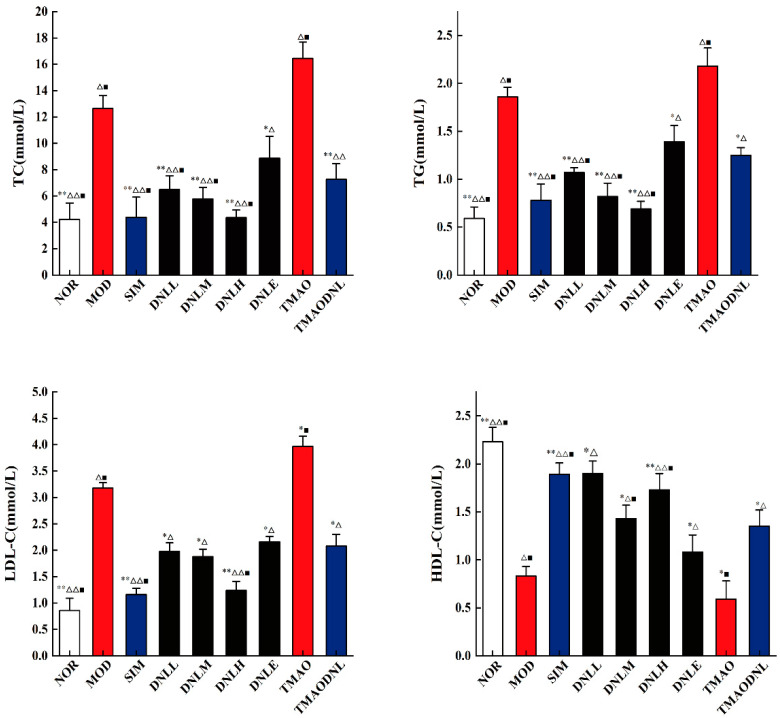
Lipid level of mice in each experimental group. vs MOD group, ** *p* < 0.01, * *p* < 0.05; vs. TMAO group, ^△△^ *p* < 0.01, ^△^ *p* < 0.05. vs. DNLE group, ^■^ *p* < 0.05.

After two weeks of medication administration, as depicted in Figure 3, mice in the MOD group had serum levels of TG, TC, and LDL-C that were very significantly higher (*p* < 0.01) compared to the NOR group, and HDL-C that was very significantly lower (*p* < 0.01), indicating that the modeling of hyperlipidemia had been successfully established. Mice in the DNL-L, DNL-M, and DNL-H groups had serum levels of TC (*p* < 0.01), TG (*p* < 0.01), and LDL-C (*p* < 0.05) that were significantly lower than those in the MOD group, while HDL-C was highly significantly higher than that in the MOD group (*p* < 0.05), suggesting that DNL enteric delayed-release granules could significantly reduce the elevation of TC, TG, and LDL-C, as well as the decrease of HDL-C, brought on by a high-fat diet. Furthermore, compared to the DNLE group, mice treated with the enteric delayed-release granules showed significant decrease in TC (*p* < 0.05), TG (*p* < 0.05), and LDL-C (*p* < 0.05), but significant increase in HDL-C (*p* < 0.05). This can also lessen the increase of TC, TG, LDL-C, and reduction of HDL-C brought on by TMAO, which raises TG and LDL-C, exacerbating hyperlipidemia.

#### 5.3.3. Liver Index

**Figure 4:**
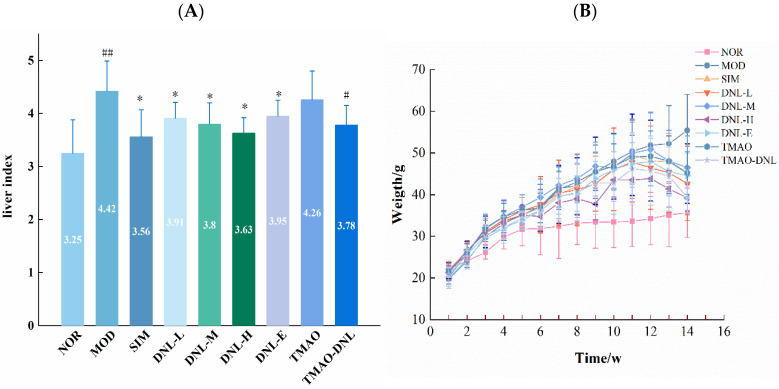
Effect of DNL on the liver index and bodyweight of mice. (**A**). Liver index of mice in each experimental group. (**B**). Changes in body mass of mice in each group. vs NOR group, ^##^ *p* < 0.01, ^#^ *p* < 0.05. vs MOD group, * *p* < 0.05.

The liver index of mice in the MOD group increased significantly (*p* < 0.01) after drug administration compared to the NOR group, as shown in Figure 4A, indicating that the mice in the MOD group had significant liver injury. No significant difference was found between the liver index of mice in the TMAO group and the MOD group (*p* > 0.05), suggesting that TMAO was unable to attenuate the liver injury in hyperlipidemic mice. Mice in the SIM, DNL-L, DNL-M, DNL-H, and DNLE group had significantly lower liver indices (*p* < 0.05), suggesting that Dendrobine and enteric delayed-release granules of Dendrobine could reduce liver injury in hyperlipidemic mice. The mice liver index of the DNL-L, DNL-M, DNL-H, and DNLE groups did not differ significantly from the SIM group (*p* > 0.05), suggesting that Dendrobine and enteric delayed-release granules of Dendrobine had the same therapeutic effect on hyperlipidemia as the positive drug Simvastatin. Additionally, the hepatic index of the mice in the TMAO-DNL group was significantly lower (*p* < 0.05) than that of the TMAO group, suggesting that DNL can mitigate TMAO-induced liver injury. 

#### 5.3.4. Body Weight

After 12 weeks of feeding, all experimental groups of mice given high-fat chow had significantly higher body weights (*p* < 0.01) than the NOR group, and there was no significant difference (*p* > 0.05) between the groups (Figure 4B). Following a 2-week administration period, mice in the NOR and MOD groups experienced an increase in body weight, while the mice in the SIM, DNL-L, DNL-M, DNL-H, DNL-E, TMAO, and TMAO-DNL groups showed a significant decrease in body weight (*p* < 0.01) when compared to the MOD group. This suggests that the sustained-release particles containing dendrobium alkaloid had the effect of delaying the increase in body weight of hyperlipidemic mice. Furthermore, the mice in the DNL-H group demonstrated a significant decrease in body weight when compared to the DNL-E group (*p* < 0.05), indicating that the effect for DNL-H group was more significant.

#### 5.3.5. Observation of Liver Morphology

**Figure 5:**
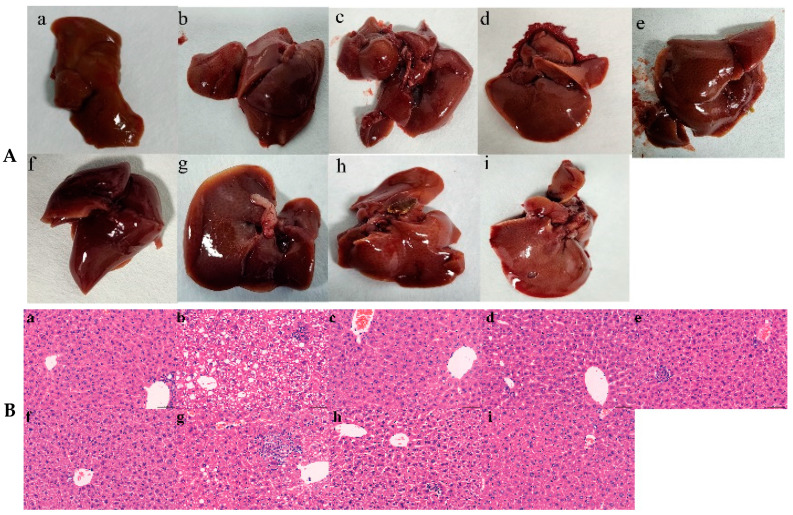
Effect of DNL on mice liver. (**A**). Observations on the appearance of the liver of mice in each experimental group; (**B**). Pathological morphology of mouse liver in each experimental group (HE, ×400, 50 μm). (**a**): Normal group. (**b**): Model group. (**c**): SIM group. (**d**): DNL-L group. (**e**): DNL-M group. (**f**): DNL-H group. (**g**): DNL-Extract group. (**h**): TMAO group. (**i**): TMAO-DNL group.

The mice livers in the NOR group were bright red and had a smooth surface, as shown in Figure 5A. The mice in the MOD group had large livers with noticeable white spots on the surface, poor liver tissue elasticity, and hemorrhagic spots. The mice in the DNL-H, DNL-M, DNL-L, SIM, and DNL-E groups were able to improve somewhat the whitish and greasy aspect of the mice with hyperlipidemia, with fewer white spots on the surface and no hemorrhagic spots. The DNL-H group improved more than the DNL-M, DNL-L, and DNL-E groups, and displayed the least amount of steatosis. The TMAO group showed an increase in the surface texture of liver tissues and the appearance of gallstones, and the DNL-TMAO group showed a slight improvement in liver tissues.

#### 5.3.6. Liver Histopathology Testing

As shown in Figure 5B, the hepatocytes of NOR group mice had normal morphology, clear nuclei, neat arrangement, microsteatosis in the cytoplasm, and the hepatic cords were radially distributed, with no pathological changes. The hepatocytes of the MOD group of mice were loosely arranged, swollen, vacuolated, with irregular morphology and a macrosteatosis, showing a severe fatty liver. The hepatocytes of the SIM group of mice had normal morphology and the macrosteatosis disappeared, while the hepatocytes of the DNL-L, DNL-M, and DNL-H groups of mice had complete, clear, and radially arranged structures, and the macrosteatosis either decreased or vanished, as demonstrated in Figure 4B. The liver cell structure and radial organization of the mice in the DNL-L, DNL-M, and DNL-H groups were complete and clear, and the macrosteatosis decreased or vanished over time, showing a mild fatty liver, indicating that enteric delayed-release granules could significantly improve fatty liver. The macro-steatosis count was significantly lower in the DNL-E group of mice than in the model group, and the mice in the TMAO group exhibited unclear margins between hepatocytes, chaotic arrangement, cytoplasmic dissolution, and vacuoles in cytoplasm. Mice in the TMAO-DNL group had an intact liver tissue structure and showed a considerable improvement in vacuolation when compared to the TMAO group.

### 5.4. 16S rRNA High-Throughput Sequencing

#### 5.4.1. Species Abundance Analysis

##### Heat Map of Species Abundance Clustering

**Figure 6:**
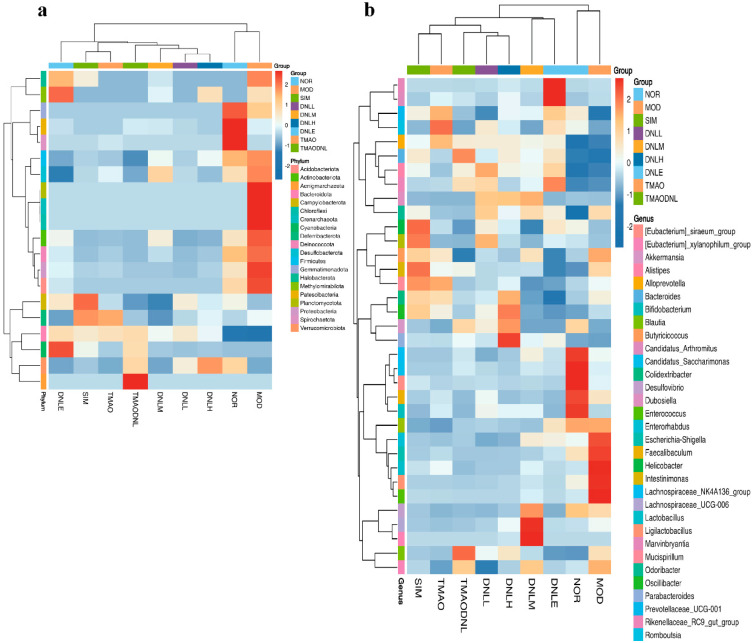
Heatmap of species abundance clustering. (**a**) The distribution of groups at level 1; (**b**) The distribution of groups at level 2.

Compared to the NOR group, the MOD group exhibited a decrease in the number of *Gemmati-monadota*, *Patescibacteria, Spirochaetota*, and *Firmicutes* species, while an increase in the number of *Halobacterota, Firmicutes* and *Desulfobacterota*, as demonstrated by the phylum level analysis displayed in Figure 6a. Compared to the MOD group, there was an increase in the number of *Planctomycetota, Crenarchaeota, Chloroflexi, Actinobacteriota, Deinococcota, Proteobacteria* and *Acidobacteriota* in the DNL-L, DNL-M, and DNL-H groups. There was an increase in the number of *Campylobacterota, Deferribacterota species, Cyanobacteria*, and *Methylo-mirabilota* species in the DNL-E group, and an increase in the number of *Deferribacterota* species in the TMAO group and TMAO-DNL group.

The genus level analysis results, as illustrated in Figure 6b, indicated an increase in the abundance of the following species: *Halobacterota*, *Firmicutes*, and *Desulfobacterota* in the MOD group; *Planctomycetota*, *Crenarchaeota*, *Chloroflexi*, *Actinobacteriota*, *Deinococcota*, *Proteobacteria*, *Acidobacteriota*, and *Campylobacterota* in the SIM group. The number of *Deferribacterota* for DNL-L, DNL-M, and DNL-H groups increased. *Desulfovibrio*, *Lachnospiraceae UCG-006*, *Weissella*, *Colidextribacter*, *Oscillibacter*, *Akkermansia*, *Verrucomicrobiota*, and *Parabacteroides* become more prevalent, There was an increase in the abundance of *Cyanobacteria*, *Marvinbryantia*, *Candidatus*, and *Arthromitus* in the DNL-E group, *Deferribacterota* in the TMAO group, and *Romboutsia*, *Aenigmarchaeota*, and *Bacteroides* in the TMAO-DNL group.

##### Petal Plots

**Figure 7:**
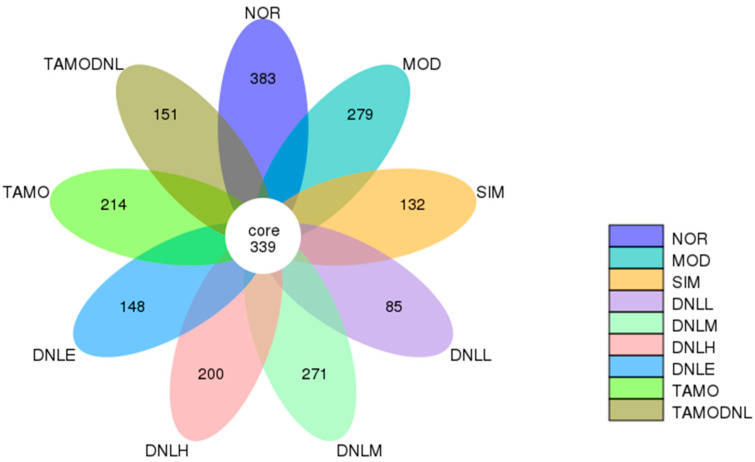
OTU Petal Chart.

At 97% similarity, the number of OTUs for the nine sample groups in this animal experiment was obtained, and the core disc map of petal OTUs was plotted. As shown in Figure 7, there were 339 OTUs in nine groups, 383 OTUs in NOR group, 279 OTUs in MOD group, 132 OTUs in SIM group, 85 OTUs in DNLL group, 271 OTUs in DNLM group, 200 OTUs in DNLH group, 148 OTUs in DNLE group, and 148 OTUs in TAMO group. TAMO group had 214 OUTs exclusively and TAMODNL group had 151 OUTs exclusively. There were differences in the degree of similarity of gut microbiota of among the groups.

##### Bar Chart of Species Abundance

**Figure 8:**
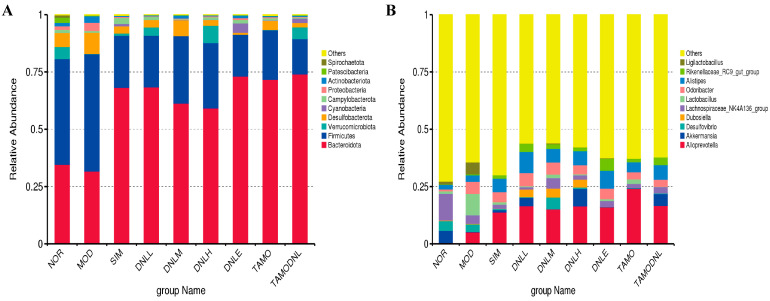
Classification results of Top 10 species with abundance in each group. (**A**) The phylum level; (**B**) The genus level.

The leading species in each experimental group were largely identical at the phylum and genus levels, as illustrated in Figure 8, but there seemed to be a larger variation in relative abundance. *Firmicutes*, *Bacteroidota*, *Verrucomicrobiota*, *Proteobacteria*, *Desulfobacterota*, *Cyanobacteria*, *Campylobacterota*, *Patinobacteria*, *Actinobacteriota*, and *Spirochaetota* displayed the top 10 relative abundance contents when phylum-level analysis was performed (Figure 8A). *Alloprevotella*, *Akkermansia*, *Desulfovibrio*, *Dubosiella*, *Lachnospiraceae_NK4A136_group*, *Odoribacter*, *Alistipes*, *Rikenellaceae_RC9_gut*, *Ligilactobacillus*, and *Lactobacillus* were the top 10 relative abundance contents when examined at the genus level (Figure 8B).

Compared with the normal group, the number of beneficial bacteria, such as *Micrococcus intestinalis* and *Ackermannia* spp. in the model group, decreased, while the number of harmful bacteria, such as *Aspergillus* spp. and *Lactobacillus* spp., increased. The number of beneficial bacteria, such as *Micrococcus intestinalis* and *Ackermannia* spp., in the hyperlipidemic mouse increased, and the number of harmful bacteria, such as *Aspergillus* spp. and *Lactobacillus* spp., decreased after treatment with the DNL enteric delayed-release granules.

##### Species Evolutionary Tree at Genus Level

**Figure 9:**
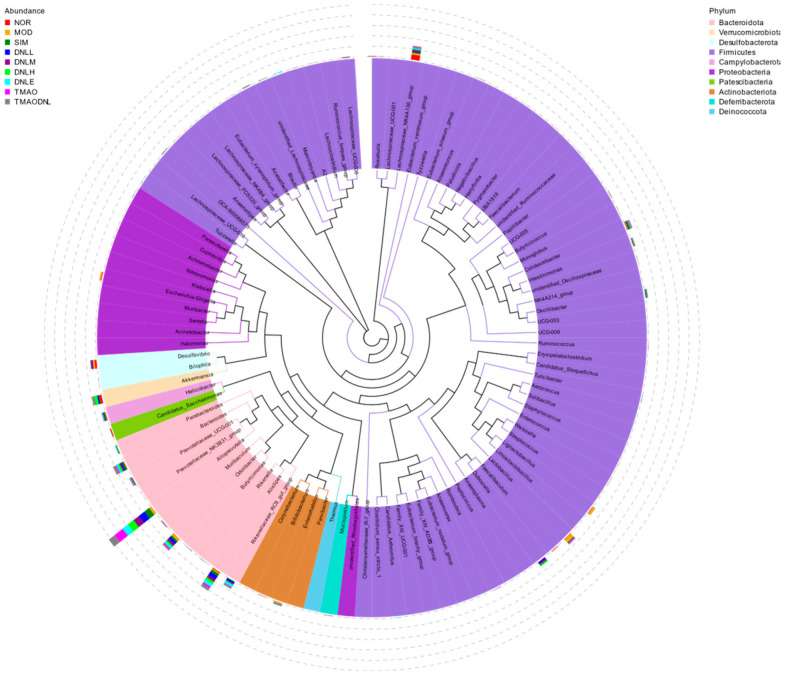
Genus level evolutionary tree.

As shown in Figure 9, representative sequences of the top 100 genera were obtained by multiple sequence comparison and found to be concentrated in *Bacteroidota*, *Verrucomicrobiota*, *Desulfobacterota*, *Firmicutes*, *Campylobacterota*, *Proteobacteria*, *Patescibacteria*, *Actinobacteriota*, *Deferribacterota*, and *Deinococcota*, in agreement with the results of the clustering analysis of species abundance.

#### 5.4.2. Alpha Diversity Analysis

##### Species Diversity Analysis

**Figure 10:**
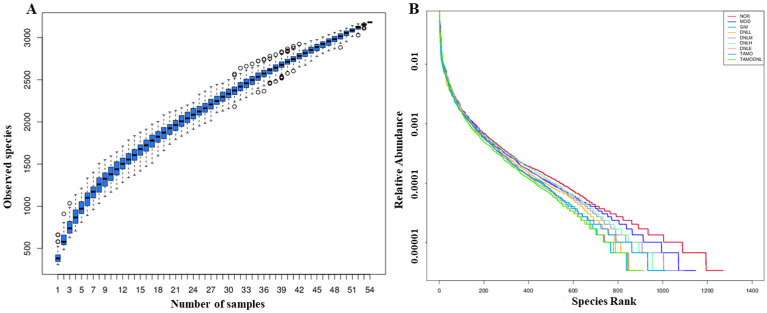
Species accumulation curves and hierarchical clustering curves. Note: (**A**): species cumulative curve. (**B**): hierarchical clustering curve.

According to the species accumulation curve in Figure 10A, it tends to flatten with the increase in sample size, indicating that the sampling is sufficient; from Figure 10B, the rank clustering curve is wide and flat, indicating that the species composition is rich and well homogenized, and the amount of sequencing data is sufficient to reflect the species diversity in the samples. Compared with the MOD group, the NOR group had a larger range on the horizontal axis and a gentler slope than the remaining groups, suggesting that the NOR group had higher species abundance and a uniform species distribution, while the MOD group curve had a smaller range on the horizontal axis and a steeper slope, suggesting that the abundance of the species in this group was reduced, and that the uniformity of species distribution was relatively low. Compared with the MOD group, the DNL-L, DNL-M and DNL-H groups had a higher abundance of species and a uniform species distribution, while the other groups had a lower abundance of species and a uniform species distribution.

##### Alpha Diversity Index

**Figure 11:**
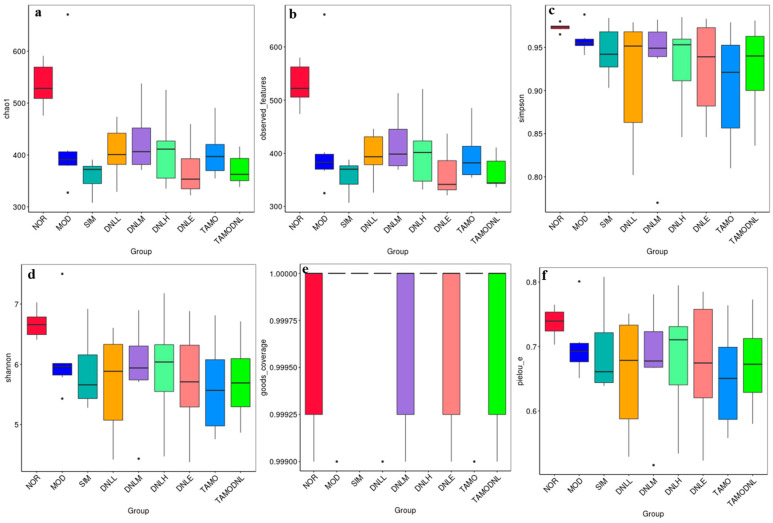
Comparison between groups via ASV diversity index boxplot diagram. Note: (**a**): Chao1; (**b**): Observed features; (**c**): Shannon index; (**d**): Simpson index; (**e**): Goods coverage; (**f**): Pielou-e.

Compared with the NOR group, the Chao1 index was significantly reduced, indicating a highly significant reduction in species in all experimental groups (*p* < 0.01). Observed features reflected that species abundance was significantly reduced in the MOD group, DNL-L group, DNL-M group, DNL-H group, DNL-E group, TMAO group, and TMAO-DNL group (*p* < 0.01), and the dendrobium base enteric delayed-release granules, simvastatin, and Dendrobine extract reduced species diversity to some extent. Shannon and Simpson indices were used to evaluate the diversity of community distributions. These suggested that the MOD group, DNL-L group, DNL-M group, DNL-H group, DNL-E group, TMAO group, TMAO-DNL group, TMAO-DNL group, TMAO-DNL group, and TMAO-DNL group had significantly (*p* < 0.01) lower species abundance. DNL group decreased in community diversity, and Pielou-e suggested that the species in each group were evenly distributed. Goods coverage indicated that the coverage of sequenced samples was high and the confidence of sequencing results was high. The diversity indices of different samples were calculated at this depth, summarized in Table 1, and plotted in the alpha diversity-related boxplot analysis (Figure 11).

Compared with the NOR group, the Chao1 index, Observed features and Shannonindex of SIM, DNL-L, DNL-H and DNL-E group, TMAO group, and TMAO-DNL group were significantly reduced (*p* < 0.05), and the Simpson index showed no significant change.

**Figure 12:**
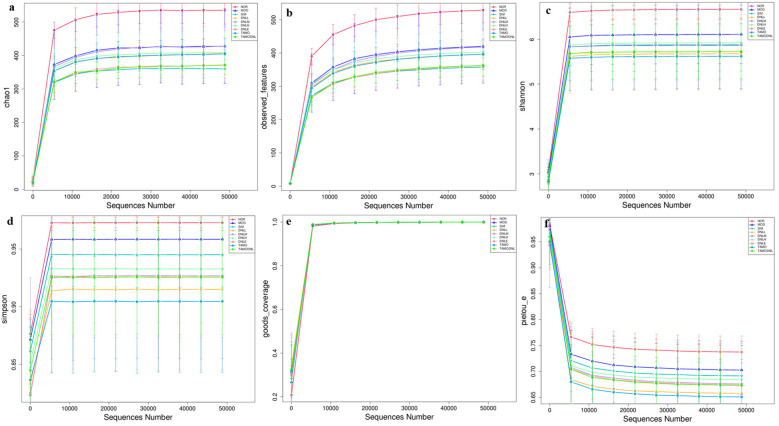
Each exponential dilution curve. (**a**) Chao1. (**b**) Observed features. (**c**) Shannon index (**d**) Simpson index. (**e**) Goods coverage. (**f**) Pielou-e.

The dilution curves for Chao1, Observed features, Shannon, Simpson, Goods coverage, and Pielou-e indices for each group of samples tend to flatten out, showing that the current sequencing results remain stable, indicating that the samples selected for this study captured most of the species. In summary, the above results, combined with Figure 12, suggest that the amount of sequencing data in this study is sufficient for subsequent analysis.

#### 5.4.3. Beta Diversity Analysis

**Figure 13:**
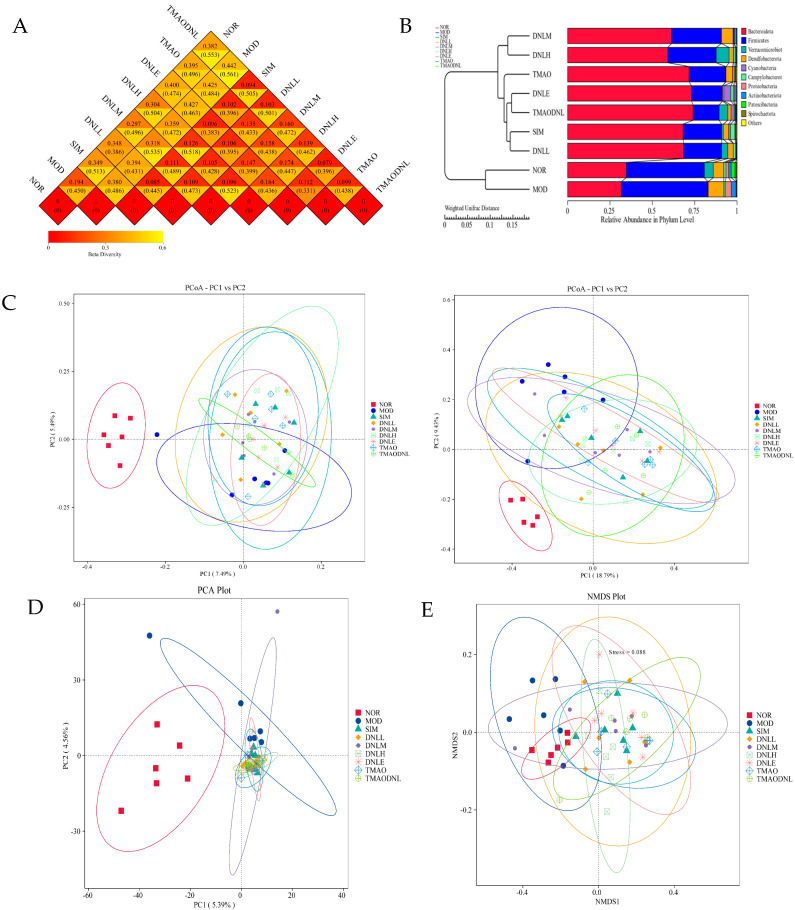
The Beta Diversity Analysis (**A**): Distance matrix heat map; (**B**): UPGMA cluster analysis; (**C**): PCoA analysis; (**D**): PCA analysis; (**E**): NMDS analysis.

The results for the distance matrix heat map(Figure 13A), UPGMA clustering analysis, PCoA analysis, PCA analysis, and NMDS analysis showed that the microbial communities of the NOR and MOD groups were significantly separated, indicating that the high-fat diet altered the species abundance and species diversity of the flora, and that the administration of simvastatin and DNL enteric delayed-release pellets reduced the differences in species diversity of the flora, suggesting that simvastatin and DNL enteric delayed-release pellets may alter the composition of intestinal flora in hyperlipidemic mice (Figure 13B). DNL was also found to reduce the differences in species diversity caused by TMAO (Figure 12a). The DNL-L, DNL-M, DNL-H, and DNL-E groups had more overlapping parts, suggesting that these groups were more similar in terms of the species composition of the intestinal flora of mice (Figure 13C).

#### 5.4.4. Statistical Testing

##### Anosim Analysis

Statistical tests showed that the NOR group was highly significantly different from all other experimental groups (*p* < 0.01), and the MOD group was significantly different from the SIM, DNL-L, DNL-M, DNL-H, DNL-E, TMAO, and TMAO-DNL-groups (*p* < 0.01, *p* < 0.05), as shown in Table 2, indicating that the between-group differences were significantly greater than the within-group differences, thus determining that the grouping was meaningful.

##### LEfSe Analysis

**Figure 14:**
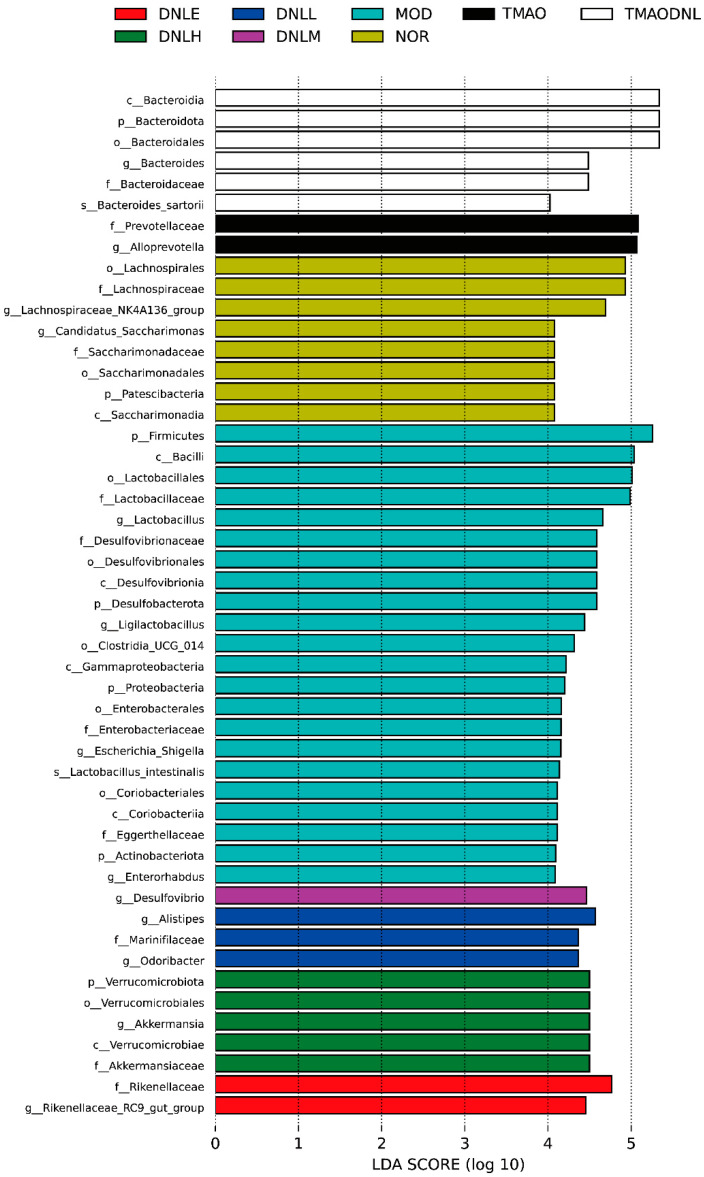
LDA discrimination from phylum to genus level.

LEfSe analysis is an analytical tool for discovering and interpreting biomarkers (genes, pathways, taxonomic units, etc.) for high-dimensional data. It emphasizes statistical significance and biological relevance, enabling comparisons between multiple subgroups and comparative subgroup analyses to find species that differ significantly in abundance between groups [11]. Biomarkers with significant differences in abundance at the phylum-to-species level were further identified for each group, as shown in Figure 14. The NOR group had eight significantly differentiated bacteria, in the order of_*Lachnospirales*, *f_Lachnospiraceae*, *g_Lachnospiraceae_NK4A136_group*, *g_Candidatus_Saccharimonas*, *f_Saccharimonadaceae*, *o_Saccharimonadales*, *p_Patescibacteria*, *c_Saccharimonadia*. The MOD group had 22 species of significantly different bacteria, mainly *p_Firmicutes*, *c_Bacilli*, *o_Lactobacillales*, *f_Lactobacillaceae*, *g_Lactobacillus*, *f_Desulfovibrionaceae*, *o_Desulfovibrionales*, *c_Desulfovibrionia*, *p_Desulfobacterota*, *g_Ligilactobacillus*, *o_Clostridia_UCG_014*, *c_Gammaproteobacteria*, *p_Proteobacteria*, *o_Enterobacterales*, *f_Enterobacteriaceae*, *g_Escherichia_Shigella*, *s_Lactobacillus_intestinalis*, *o_Coriobacteriales*, *c_Coriobacteriia*, *f_Eggerthellaceae*, *p_Actinobacteriota*, and *g_Enterorhabdus*; the DNL-L group after the drug intervention had three significantly different bacteria, in the order of *g_Alistipes*, *f_Marinifilaceae*, and *g_Odoribacter;* and the DNL-M group had one significant bacteria, *g_Desulfovibrio*; the DNL-H group had five species of significantly different bacteria, in the order of *p_Verrucomicrobiota*, *o_Verrucomicrobiales*, *g_Akkermansia*, *c_Verrucomicrobiae*, *f_ Akkermansiaceae*; The DNL-E group had two significantly different bacteria, *f_Rikenellaceae*, and *g_Rikenellaceae_RC9_gut_group*; The TMAO group had two significantly different bacteria, *f_Prevotellaceae* and *g_Alloprevotella*; The TMAO-DNL group had six differentially significant bacteria, in order of *c_Bacteroidia*, *p_Bacteroidota*, *o_Bacteroidales*, *g_Bacteroides*, *f_Bacteroidaceae*, and *s_Bacteroides_sartorii*.

**Figure 15:**
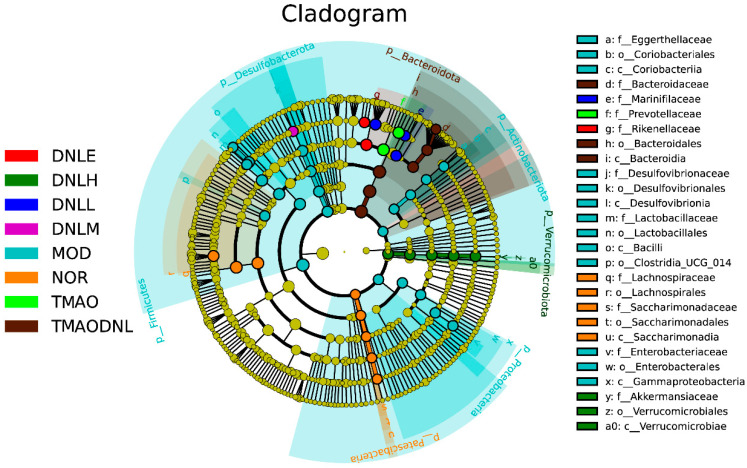
LEfSe analysis at the gate-to-genera level.

In the evolutionary branching diagram (Figure 15), the dominant bacterial groups in the NOR group were *f_Lachnospiraceae*, *o_Lachnospirales*, *f Saccharimonadaceae*, *o_Saccharimonadales*, and *c_Saccharimonadia*, and the dominant bacterial groups in the MOD group role were *f_Eggerthellaceae*, *o_Coriobacteriales*, *c_Coriobacteriia*, *f_Desulfovibrionaceae*, *o_Desulfovibrionales*, *c_Desulfovibrionia*, *f_Lactobacillaceae*, *o_Lactobacillales*, *c_Bacilli*, *o_Clostridia_UCG_014*, *f_Enterobacteriaceae*, *o_Enterobacterales*, and *c_Gammaproteobacteria*. The dominant bacterial group in the TMAO group was *f_Prevotellaceae*, the dominant bacterial group in the DNL-E group was *f_Rikenellaceae*, and the dominant bacterial group in the DNL-L group was *f_Marinifilaceae* and the dominant bacterial group in the DNL-H group was *f_Akkermansiaceae*, *o_Verrucomicrobiales*, *c_Verrucomicrobiae*. The dominant flora in the TMAO-DNL group were *f_Bacteroidaceae*, *o_Bacteroidales* and *c_Bacteroidia*.

To summarize the results, the number of species/differentially significant colonies in the NOR group was much smaller than that in the MOD group, and the number of species/differentially significant colonies in the MOD group increased, indicating that elevated lipids led to large changes in the dominant colonies of the bacterial flora, and the reduction of species/differentially significant colonies after the administration of simvastatin and DNL interventions indicated that simvastatin and DNL could restore the diversity of the colonies’ species to a certain degree, and possibly through a role played by *g_Alistipes*, *f_Marinifilaceae*, *g_Odoribacter*, *g_Desulfovibrio*, *p_Verrucomicrobiota*, *o_Verrucomicrobiales*, *g_Akkermansia*, *c_Verrucomicrobiae* and *f_Akkermansiaceae*.

#### 5.4.5. Tax4Fun Functionality Predictive Analytics

##### Relative Abundance Cluster Analysis

**Figure 16:**
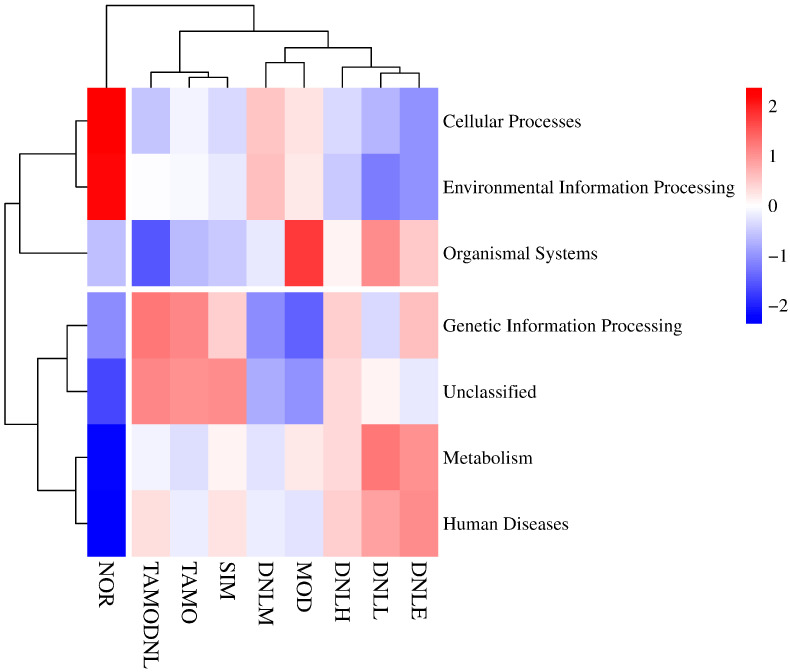
Distribution of groups at level 1.

As seen in Figure 16, KEGG functional annotation based on Tax4Fun was used to further evaluate microbial community alterations, which found the most closely related sequences by comparing 16S rRNA gene sequences to reference sequences and then predicted the function of microbial communities based on the functional profiles of these sequences. At level 1, the correlation heatmap derived from enriching the functional genes of the samples in each group revealed that the functional genes of each group were primarily enriched in cellular processes, human diseases, and organic systems. In comparison to the NOR group, the abundance of genes related to cellular processes was less abundant and functionally down-regulated in the other experimental groups, while the abundance of genes related to organic systems was more abundant and functionally up-regulated in the MOD group. However, the administration of Simvastatin treatment reduced the abundance of organic system-related genes and down-regulated their functions, and DNL treatment up-regulated the activities of metabolism-related genes and increased their abundance, indicating that DNL may have an impact on the intestinal flora’s metabolism in mice. In contrast, the TMAO and TMAO-DNL groups’ gene expression was primarily concentrated in unknown functions and genetic information processing.

**Figure 17:**
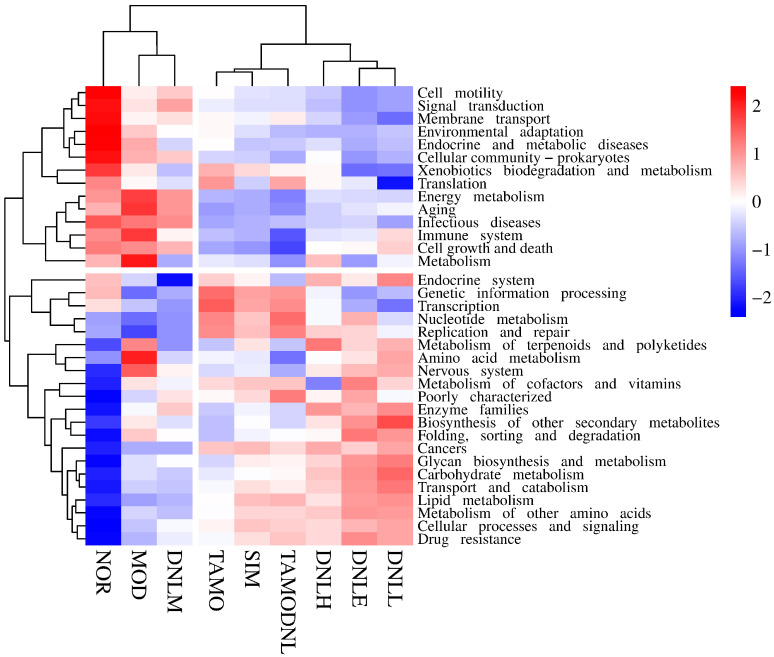
Distribution of groups at level 2.

Functional genes were primarily enriched in global and overview mapping and metabolic pathways (carbon metabolism, cofactor and vitamin metabolism, glycan biosynthesis and metabolism) at Level 2 (Figure 17). The MOD group had more enriched abundance and up-regulated functional genes in terpene and polyketide metabolism, amino acid metabolism, and neurological functions than the NOR group. The SIM group’s functional genes were primarily enriched in transcription and gene information processing. The MOD group had lower enriched abundance and down-regulated functional genes in membrane transport, signal transduction, cell motility, cell community-prokaryotes, endocrine and metabolic disorders, and environmental adaptations. The TMAO group and the TMAO-DNL group primarily showed enriched gene expression in replication and repair, nucleotide metabolism, transcription, and genetic information processing, while the DNL-L group showed up-regulation of functional genes for biosynthesis of other secondary organisms, the DNL-M group showed up-regulation of functional genes for infectious diseases, energy metabolism and aging, and the DNL-H group showed up-regulation of functional genes for metabolism of terpenoids and polyketides.

**Figure 18:**
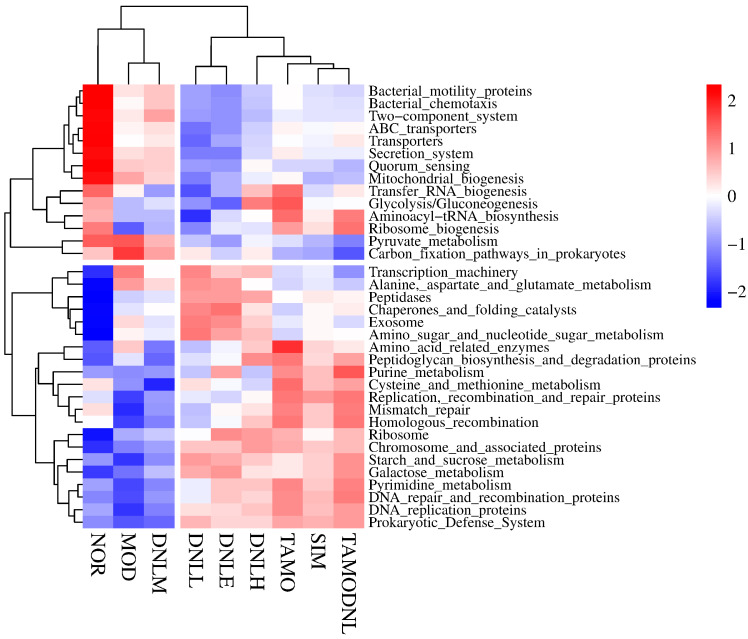
Distribution of groups at level 3.

At the LEVEL 3 level (Figure 18), the NOR group is functionally enriched in mitochondrial biogenesis, quorum sensing, two-component systems, bacterial motor proteins, bacterial chemotaxis, secretion systems, transporters, ABC transporters, and up-regulated functionally; the MOD group is functionally enriched in the carbon fixation pathway in prokaryotes, and up-regulated functionally; the SIM group is up-regulated in the genes for replication, recombination and repair functions; in the DNL-L group, Peptidase, chaperonin and folding catalyst, exosome, amino acid sugar and nucleotide sugar metabolism, alanine, aspartate and glutamate metabolism, and transcriptional machinery functional genes were up-regulated; in the DNL-M group, enriched carbon fixation pathways in prokaryotes were functionally up-regulated; In the DNL-H group, glycolysis/gluconeogenesis, peptidoglycan biosynthesis and degradation genes enriched in glycolysis were functionally up-regulated; in the TMAO group, amino acid related enzymes, peptidoglycan biosynthesis, degradation of protein purine metabolism, cysteine and methionine metabolism, replication, recombination and repair, protein mismatch repair, and homologous recombination were functionally enriched and increased; in the TMAO-DNL group, pyruvate metabolism, mitochondrial biogenesis, quorum sensing, the two-component system, aminoacyl-tRNA biosynthesis, and ribosome biogenesis were functionally enriched.

Analysis of these pathways revealed that starch and sucrose metabolism, fructose and mannose metabolism, galactose metabolism, lipid metabolism, and glycerophospholipid metabolism associated with hyperlipidemia changed significantly after DNL intervention in the intestinal flora, suggesting that lipid-lowering by DNL is associated with the modulation of glucose metabolism and lipids.

**Figure 19:**
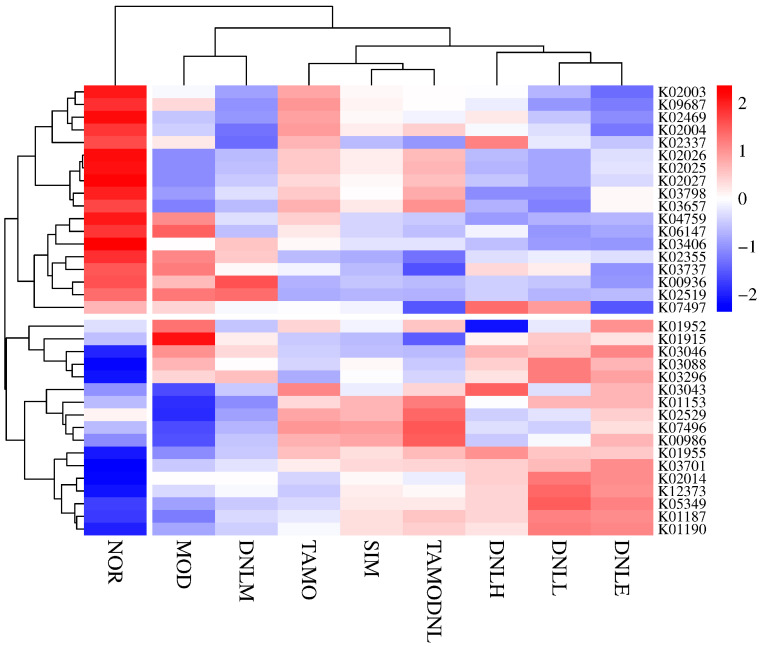
Distribution of groups at KO entry level.

At the KO entry level (Figure 19), NOR group functions are enriched in k02025, k02026, k02027, k03657, k03798, k02337, k02003, k09687, k02004, k02469, k03406, k06147, k04759, k00936, k02519, k02355, k03737; MOD group functionally enriched in k01915, k01952, k06147, k04759, k02519, k02355, k03737; SIM group functionally enriched in k00986, k07496; DNL-L group functionally enriched in k03296, k03088, k02014, k12373, k05349, k01190, k01187; DNL-M group functionally enriched at k00936, k02519; DNL-H group functionally enriched at k03043, k01955, k07497, k02337; TMAO group functionally enriched at k03043, k01153, k02529, k00986, k07496; TMAO-DNL group functionally enriched in k01153, k02529, k00986, k07496, k03657.

Analysis of these pathways revealed glycolysis/gluconeogenesis, peptidoglycan biosynthesis and degradation, pyruvate metabolism, and mitochondrial biogenesis ribosome biogenesis associated with hyperlipidemia after DNL intervention in the gut flora.

In summary, correlation heat map analysis revealed that functional genes in each experimental group given DNL were mainly enriched in metabolic pathways at level 1, level 2, level 3, and k0, suggesting that Dendrobine enteric delayed-release particles may be used to reduce lipid levels in mice by modulating intestinal flora.

##### PCA Analysis

**Figure 20:**
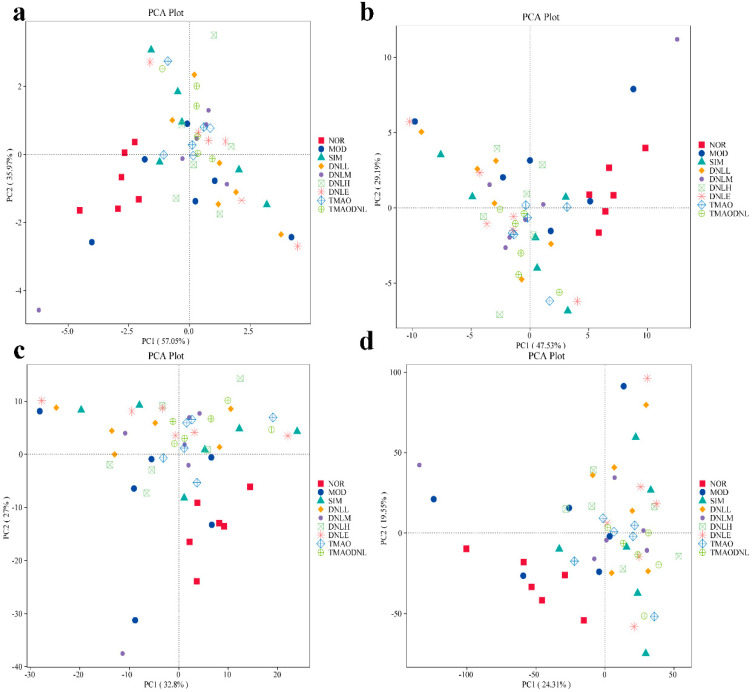
PCA charts for each level. (**a**) The PCA chart of pathway level 1functional gene. (**b**) The PCA chart of pathway level 2 functional gene. (**c**) The PCA chart of pathway level 3 functional gene. (**d**) The PCA chart of K0 functional gene.

As shown in Figure 20, for each group at level 1 (20-a), level 2 (20-b), level 3 (20-c), and level k (20-d), the NOR group and MOD group were both farther apart in the downscaling diagram, which indicated that the functional compositions of the two groups were more different, whereas the SIM group, the DNL-L group the DNL-M group, the DNL-H group, the DNL-E group, the TMAO group, and the TAML-DNL group were closer to one another in the downscaling diagram, which indicated that their functional compositions were more similar. The closer distance in the descending plot indicated that their functional composition was more similar, consistent with the results of species analysis.

## 6. Discussion

It has been discovered that a high-fat diet can cause intestinal flora imbalance, and intestinal flora is frequently dysbiotic in hyperlipidemic individuals [12]. Flora intervention via probiotic supplementation or medication can prevent the reproduction of bad bacteria while increasing the synergistic proliferation of beneficial bacteria [13,14]. Probiotics can nutritionally repair injured intestinal mucosa, restore intestinal mucosal barrier function, and prevent flora displacement [15,16]. The results of this experimental study demonstrated that Dendrobine considerably impacted the shape and function of intestinal flora in high-fat diet-fed mice. The dominating intestinal flora altered by Dendrobine were highly connected with lipid metabolism, suggesting that Dendrobine may be able to reduce the lipid metabolism in mice induced by high-fat diet by regulating the structure and function of intestinal flora. It was found that enteric delayed-release pellets made of Dendrobine could not only reduce the body weight, TC, TG, and LDL-C levels in blood lipids, and elevate HDL-C level in hyperlipidemic mice, but also significantly alter the structure and abundance of the intestinal flora in mice. In addition, our results also showed that the hypolipidemic effect of enteric delayed-release granules was significantly stronger than that of Dendrobine.

16 Sr RNA sequencing of mouse intestinal flora, PCoA analysis based on Jaccard and Bray–Curtis, and UPGMA analysis based on Weighted Unifrac distance matrix revealed that the structural distribution of intestinal flora in each group of mice was clearly clustered. At the phylum level, the high-fat diet significantly increased the warty microflora phylum of the intestinal flora of mice, whereas simvastatin administration significantly increased the *Actinobacteria phylum*, the *Thick-walled phylum*, and decreased the *Anaplasma phylum*, and DNL administration increased the *Actinobacteria phylum, Desulfovibrio phylum, the Thick-walled phylum,* and decreased the *Anaplasma phylum* and the *Cyanobacteria phylum*. When TMAO was administered, the *anaplasmosis phylum* increased extremely significantly, while the *thick-walled* and *actinomycete phylums* decreased significantly. When DNL was administered, the *anaplasmosis phylum* increased significantly, and the *thick-walled* and *actinomycete phylums* significantly decreased. Additionally, the desulfurization of the *Vibrio phylum* appeared to be significantly reduced, which is consistent with the previous administration of DNL, which also showed a significant reduction in the desulfurization of the *Vibrio phylum*. The lack of significance (*p* > 0.05) in the strain differences between the TMAO and TMAO-DNL groups was hypothesized to be attributable to either an inadequate DNL administration dose or an insufficient administration time.

At the phylum level, we hypothesize that the characteristic flora of hyperlipidemic disorders may comprise Micrococcus wartyi, while the characteristic flora of dendrobium alkaloid-regulated diseases may include *Actinobacteria, Thickettsia, Desulfovibrio, Anaplasma*, and *Cyanobacteria*. At the genus level, we hypothesize that *Lactobacillus, Alternaria, Ricinobacter butyric acid, Prevotella, Mycobacterium* and *Paramycobacterium* may be the characteristic groups of dendrobium alkaloid-regulated flora, and that *Ackermannia, Trichosporonaceae, Prevotella, Rhombus* and *Ruminalococcus* may be the characteristic genera of hyperlipidemic diseases.

Our study revealed that the number of *Firmicutes* and *Bacteroidetes* was higher in the NOR and DNL groups. Following the administration of the DNL to hyperlipidemia mice, the relative abundance of *Firmicutes* decreased, and the proportion of *Firmicutes/Bacteroidetes* decreased. Therefore, the DNL may affect the composition of intestinal flora by reducing the relative abundance of *Firmicutes* and increasing the relative abundance of *Bacteroidetes*. At the same time, after the administration of the DNL in hyperlipidemia mice, *Bacteroidales_unclassified* was up-regulated and *Streptococcus* was down-regulated, which were considered as the flora closely related to hyperlipidemia. *Bacteroidales_unclassified* belongs to the butyrate-producing bacteria, a high-fat diet can induce the decrease of intestinal flora to lead to the decrease of butyrate, the decrease of butyrate will destroy the balance of pH value in the living environment of intestinal flora, and butyrate can promote cholesterol transport by increasing the mRNA level and the secretion of phospholipid transporters. Streptococcus can increase the production of endotoxin to aggravate the inflammatory reaction, leading to dyslipidemia. These results suggest that the DNL can regulate lipid metabolism mainly by up-regulating *Bacteroidales_unclassified*, and down-regulating the harmful bacterium *Streptococcus* in hyperlipidemia mice.

The functional genes of dendrobium alkaloids were found to be enriched in metabolic pathways at level 1, level 2, level 3, and level k0 level differences, according to Tax4Fun functional prediction analysis. This suggests that dendrobium alkaloids improve blood lipid levels by modulating the characteristic flora in life processes, such as energy metabolism, terpenoid and polyketide compounds, aging, messenger RNA biogenesis, starch and sucrose metabolism, etc. Additionally, k01153 (type I restriction enzyme, R subunit), k01154 (type I restriction enzyme, S subunit), k03555 (DNA mismatch repair protein MutS), k07456 (DNA mismatch repair protein MutS2), and k04485 (DNA repair protein RadA/Sms) (methionyl-tRNA synthetase), k07462 (single-stranded DNA-specific exonuclease), and others are linked to an increase in functional enrichment.

In summary, we found that hyperlipidemia leads to Thick-walled Bacteria, Bacillus, Lactobacillus, Lactobacillus, *Lactobacillus* spp., Desulfovibrio, Desulfovibrio, Desulfovibrio, Desulfovibrio, Desulfovibrio, *Lactobacillus* spp., *Lactobacillus* spp. united, o_Clostridia_UCG_014, γ-Amoebicans, Ascomycetes, Enterobacteriaceae, Enterobacteriaceae, g_Escherichia_ Shigella, s_Lactobacillus_intestinalis, erythrobacteriophage, erythrobacteriophage, f_Eggerthellaceae, Actinobacteriaceae, Enterobacteriaceae, etc., and DNL could significantly improve the intestinal flora disorder, restore the diversity and abundance of intestinal flora in mice, and accelerate the recovery of hyperlipidemia-induced intestinal flora disorders in mice. 

## Figures and Tables

**Table 1:** Alpha diversity index.

Groops	Chao1 Index	Observed Features	Shannon Index	Simpson Index	Goods Coverage	Pielou-e
NOR	534.81 ^△△^	529.00 ^△△^	6.67 ^△^	0.97	1.00	0.74
MOD	427.93 **	420.17 **	6.11	0.96	1.00	0.70
SIM	359.87 **^△△^	356.67 **^△△^	5.87 ^△^	0.95	1.00	0.69
DNL-L	405.89 **^△^	396.00 **^△^	5.68 *	0.92	1.00	0.66
DNL-M	427.07 **	418.17 **	5.88 ^△^	0.93	1.00	0.68
DNL-H	408.65 **^△^	402.67 **^△^	5.92 ^△^	0.93	1.00	0.68
DNL-E	370.56 **^△^	361.67 **^△△^	5.72 *^△^	0.93	1.00	0.67
TMAO	404.79 **	396.50 **^△^	5.62 *^△^	0.90 *	1.00	0.65
TMAO-DNL	371.64 **^△^	362.83 **^△^	5.72 *^△^	0.93	1.00	0.67

Note: vs. NOR group,* *p* < 0.05; ** *p* < 0.01. Note: vs. MOD group, ^△^ *p* < 0.05; ^△△^ *p* < 0.01.

**Table 2:** Comparison of differences between groups.

Group1	Group2	R-Value	*p*-Value
NOR	MOD	0.74815	0.007
NOR	SIM	0.90741	0.003
NOR	DNL-L	0.75	0.004
NOR	DNL-M	0.62778	0.003
NOR	DNL-H	0.92778	0.004
NOR	DNL-E	0.88333	0.003
NOR	TMAO	0.95185	0.004
NOR	TMAO-DNL	0.94815	0.003
MOD	SIM	0.55185	0.006
MOD	DNL-L	0.5463	0.004
MOD	DNL-M	0.31296	0.025
MOD	DNL-H	0.70926	0.004
MOD	DNL-E	0.88333	0.003
MOD	TMAO	0.95185	0.004
MOD	TMAO-DNL	0.94815	0.003
TMAO	TMAO-DNL	0.02222	0.343

## Data Availability

The raw data supporting the conclusions of this article will be made available by the authors upon request.

## References

[1] Alloubani A., Nimer R., Samara R. (2021). Relationship between Hyperlipidemia, Cardiovascular Disease and Stroke: A Systematic Review. Curr. Cardiol. Rev..

[2] Jia X., Xu W., Zhang L., Li X., Wang R., Wu S. (2021). Impact of Gut Microbiota and Microbiota-Related Metabolites on Hyperlipidemia. Front. Cell. Infect. Microbiol..

[3] Nishida A., Ando Y., Kimura I., Miyamoto J. (2022). Involvement of Gut Microbial Metabolites Derived from Diet on Host Energy Homeostasis. Int. J. Mol. Sci..

[4] Vourakis M., Mayer G., Rousseau G. (2021). The Role of Gut Microbiota on Cholesterol Metabolism in Atherosclerosis. Int. J. Mol. Sci..

[5] Xia M., Xu Y., Li H., Huang J., Zhou H., Gao C., Han J. (2024). Structural and functional alteration of the gut microbiota in elderly patients with hyperlipidemia. Front. Cell. Infect. Microbiol..

[6] Fan C., Sun X., Wang X., Yu H. (2023). Therapeutic potential of the chemical composition of Dendrobium nobile Lindl. Front. Pharmacol..

[7] Feng Y., Jia B., Feng Q., Zhang Y., Chen Y., Meng J. (2021). Dendrobine attenuates gestational diabetes mellitus in mice by inhibiting Th17 cells. Basic. Clin. Pharmacol. Toxicol..

[8] Kim Y.R., Han A.R., Kim J.B., Jung C.H. (2021). Dendrobine Inhibits γ-Irradiation-Induced Cancer Cell Migration, Invasion and Metastasis in Non-Small Cell Lung Cancer Cells. Biomedicines.

[9] Li R., Liu T., Liu M., Chen F., Liu S., Yang J. (2017). Anti-influenza A Virus Activity of Dendrobine and Its Mechanism of Action. J. Agric. Food Chem..

[10] Li Y.H., Jiang Z.X., Xu Q., Jin T.T., Huang J.F., Luan X., Li C., Chen X.Y., Wong K.H., Dong X.L. (2024). Inhibition of calcium-sensing receptor by its antagonist promotes gastrointestinal motility in a Parkinson’s disease mouse model. Biomed. Pharmacother..

[11] Yu X., Jiang W., Kosik R.O., Song Y., Luo Q., Qiao T., Tong J., Liu S., Deng C., Qin S. (2021). Gut microbiota changes and its potential relations with thyroid carcinoma. J. Adv. Res..

[12] Xu D., Feng M., Chu Y., Wang S., Shete V., Tuohy K.M., Liu F., Zhou X., Kamil A., Pan D. (2021). The Prebiotic Effects of Oats on Blood Lipids, Gut Microbiota, and Short-Chain Fatty Acids in Mildly Hypercholesterolemic Subjects Compared with Rice: A Randomized, Controlled Trial. Front. Immunol..

[13] Liu M., Shi W., Huang Y., Wu Y., Wu K. (2023). Intestinal flora: A new target for traditional Chinese medicine to improve lipid metabolism disorders. Front. Pharmacol..

[14] Xu F., Yu Z., Liu Y., Du T., Yu L., Tian F., Chen W., Zhai Q. (2023). A High-Fat, High-Cholesterol Diet Promotes Intestinal Inflammation by Exacerbating Gut Microbiome Dysbiosis and Bile Acid Disorders in Cholecystectomy. Nutrients.

[15] Wang S., Ren H., Zhong H., Zhao X., Li C., Ma J., Gu X., Xue Y., Huang S., Yang J. (2022). Combined berberine and probiotic treatment as an effective regimen for improving postprandial hyperlipidemia in type 2 diabetes patients: A double blinded placebo controlled randomized study. Gut Microbes.

[16] Zeng Y., Zhao J., Zhang J., Yao T., Weng J., Yuan M., Shen X. (2023). Development of a Nomogram That Predicts the Risk of Coronary Heart Disease in Patients With Hyperlipidemia. J. Cardiovasc. Pharmacol..

